# Comprehensive analysis of bZIP transcription factors uncovers their roles during dimorphic floret differentiation and stress response in *Cleistogenes songorica*

**DOI:** 10.1186/s12864-019-6092-4

**Published:** 2019-10-22

**Authors:** Qi Yan, Fan Wu, Tiantian Ma, Xifang Zong, Qian Ma, Jie Li, Yufeng Zhao, Yanrong Wang, Jiyu Zhang

**Affiliations:** State Key Laboratory of Grassland Agro-ecosystems, Key Laboratory of Grassland Livestock Industry Innovation, Ministry of Agriculture and Rural Affairs, College of Pastoral Agriculture Science and Technology, Lanzhou University, Lanzhou, 730020 People’s Republic of China

**Keywords:** *Cleistogenes songorica*, Transcription factor, *BZIP* genes, Evolutionary analysis, Cleistogamous, Stress response

## Abstract

**Background:**

Transcription factors act as important regulators of transcription networks. Basic leucine zipper (bZIP) transcription factors have been shown to be involved in multiple biological processes in plants. However, no information is available for the bZIP family in *Cleistogenes songorica,* which is an important xerophytic and allotetraploid grass in desert grasslands.

**Results:**

In this study, 86 *CsbZIPs* were identified in the allotetraploid *C*. *songorica* genome. For location analysis, *CsbZIPs* were distributed evenly across two subgenomes of *C*. *songorica*. Phylogenetic tree analysis among three species indicated that *CsbZIPs* were evolutionarily more closely related to *OsbZIPs* than *AtbZIPs*. Syntenic and phylogenetic analyses confirmed that the *CsbZIPs* were mainly expanded by whole-genome duplication events. Furthermore, it was determined that rice and *C. songorica* might have undergone purified selection during their long evolutionary history by calculating the Ks values and Ka/Ks ratios of orthologous gene pairs. By analysing the expression patterns of *CsbZIPs* in different tissues and under abiotic stresses, 21 *CsbZIP* genes were differentially expressed between chasmogamous (CH) and cleistogamous (CL) flowers, including two *FLOWERING LOCUS D* (*FD*) genes. In shoots and roots, 79.1 and 87.2% of the *CsbZIP* genes, respectively, displayed transcript changes under at least one stress treatment, such as heat, cold, drought and salt. Strikingly, 17 common *CsbZIP* genes showed differential expression under stress response and during CL flowering. Co-expression network, GO annotation and real-time quantitative reverse transcription PCR (qRT-PCR) analyses revealed a close relationship between CL flowering-associated genes and abiotic stress-related genes.

**Conclusions:**

BZIP TFs were comprehensively analysed and identified in allotetraploid *C. songorica*. Our results provide insights into the evolutionary history of the bZIP family in *C. songorica* and provide abiotic stress-responsive and CL-associated candidate *CsbZIP* genes for potential applications in the genetic improvement of plants.

## Background

Transcription factors (TFs) regulate the expression of functional genes by interacting with downstream promoter regions. The bZIP TF family is one of the largest and most conserved TF families in plants [1]. In general, the bZIP domain contains a conserved basic region and a leucine zipper region [2, 3]. The basic region is composed of a conserved N-x_7_-R/K motif within 18 amino acid residues [4, 5]. The leucine zipper region contains several leucine repeats or hydrophobic amino acids [6]. The basic region and leucine zipper region are responsible for DNA binding and specific DNA identification, respectively. Furthermore, bZIP TFs can be divided into several subfamilies based on their conserved motifs. For example, the subfamily A bZIP TFs contain abscisic acid (ABA)-responsive elements in the promotor region, named ABA-responsive element binding proteins (AREB) or AREB binding factors (ABF), which have been identified to play an important role in stress signalling [7, 8]. The subfamily S bZIP TFs respond to extreme environmental stress [9].

BZIP TFs have been demonstrated to be involved in many important biological processes in plants, such as flowering, lateral root development, biomass, lipid productivity, pollen germination and seed maturation [10–14]. For example, *FLOWERING LOCUS D* (*FD*), which encodes a bZIP transcription factor, is mainly expressed in the shoot apex and is required for *FLOWERING LOCUS T* (*FT*) to initiate flowering in *Arabidopsis thaliana* [10]. In *Arabidopsis*, *fd* mutants showed a late flowering phenotype [10]. *AtFD* overexpression caused a reduction in plant height and spikelet size in transgenic rice plants [15]. In addition, *AtbZIP34* has been characterized as involved in pollen wall patterning by controlling many metabolic pathways of lipid metabolism and/or cellular transport in *Arabidopsis* [16]. The lipid content and biomass of transgenic *Nannochloropsis salina* was increased by overexpressing *NsbZIP1* [11]. Increasing evidence has indicated that bZIP TFs play a central role in the regulation of plant responses to biotic and abiotic stress, including water deficit [17], salt toxicity [18], temperature fluctuations [19], abscisic acid (ABA), gibberellic acid signalling [20], methyl jasmonate signalling [21] and defence against pathogens [22, 23]. For instance, overexpression of *MebZIP3* and *MebZIP5* improved the disease resistance for cassava bacterial blight in tobacco [24]. In tomato, the transcription factor *SlbZIP1* is involved in salt and drought resistance by regulating ABA biosynthesis [25]. The *OsbZIP71* RNAi knockdown transgenic plants were highly sensitive to salt and polyethylene glycol stress [26]. Together, these studies showed that bZIPs are widely involved in biological processes and various stresses in plants.

Currently, bZIP TFs have been identified in numerous species, such as *Arabidopsis* [9], *Oryza sativa* [1], *Brassica napus* [27], strawberry [28], *Brachypodium distachyon* and cassava with genome sequencing [4, 5]. However, no studies have characterized and identified bZIP family members in *C. songorica,* which is one of the most important native perennial forage and ecological grasses in the desert grassland of Northwest China. *C. songorica*, an allotetraploid plant, can grow in semi-arid and desert areas where the mean annual rainfall is 110 mm [29]. Strikingly, *C. songorica* can produce both chasmogamous (CH) and cleistogamous (CL) flowers on a plant, but they appear in different locations [30]. Additionally, these two types of flowers have some differences in the morphology of their floral organs, such as lodicule size, pollen, lemma and stigma length. In *Arabidopsis* and alfalfa, overexpression of the *C. songorica LEA* and *CsALDH* genes improved the tolerance to drought and salt stress in transgenic lines [31–34]. Previously, we obtained a high-quality genome sequence and expression data under abiotic conditions for *C. songorica* (data not published). Understanding the molecular mechanisms by which the *bZIP* genes of *C. songorica* respond to abiotic stress and CL flowering may provide a valuable genetic resource for the improvement of other grasses and crops. In this study, the bZIP family was identified in the *C. songorica* genome to reveal the phylogenetic relationships, conserved motifs, gene structures, synteny, co-expression networks, and *cis-*elements of the bZIP TFs. In addition, the CL flowering and abiotic stress-related genes were also analysed.

## Results

### Genome-wide identification of bZIP family genes in *C. songorica*

In this study, 86 predicted *CsbZIP* genes were identified from the *C. songorica* genome, named *CsbZIP1* to *CsbZIP86* (Additional file 1: Table S1). Furthermore, the amino acid residues, grand average of hydropathicity (GRAVY), isoelectric points (pIs), molecular weights (Mws), and CDS lengths of the CsbZIP proteins were analysed. The 86 CsbZIP proteins varied from 122 (CsbZIP68) to 1032 (CsbZIP79) amino acid residues, the CDSs were distributed from 377 to 3175 bp, the relative Mws ranged from 13.8 (CsbZIP19) to 113.4 (CsbZIP79) KDa, the GRAVY values ranged from − 1.194 (CsbZIP30) to 0.483 (CsbZIP69), and the pIs ranged from 4.81 (CsbZIP68) to 11.7 (CsbZIP47; Additional file 1: Table S1). The analysis of subcellular location showed that 92 (95.3%) CsbZIP proteins were anchored in the nucleus. In subfamily B, two and one CsbZIP proteins (60%) were anchored in the endoplasmic reticulum and plasma membrane, respectively. In addition, CsbZIP70 were anchored in chloroplast (Additional file 1: Table S1).

**Fig. 1:**
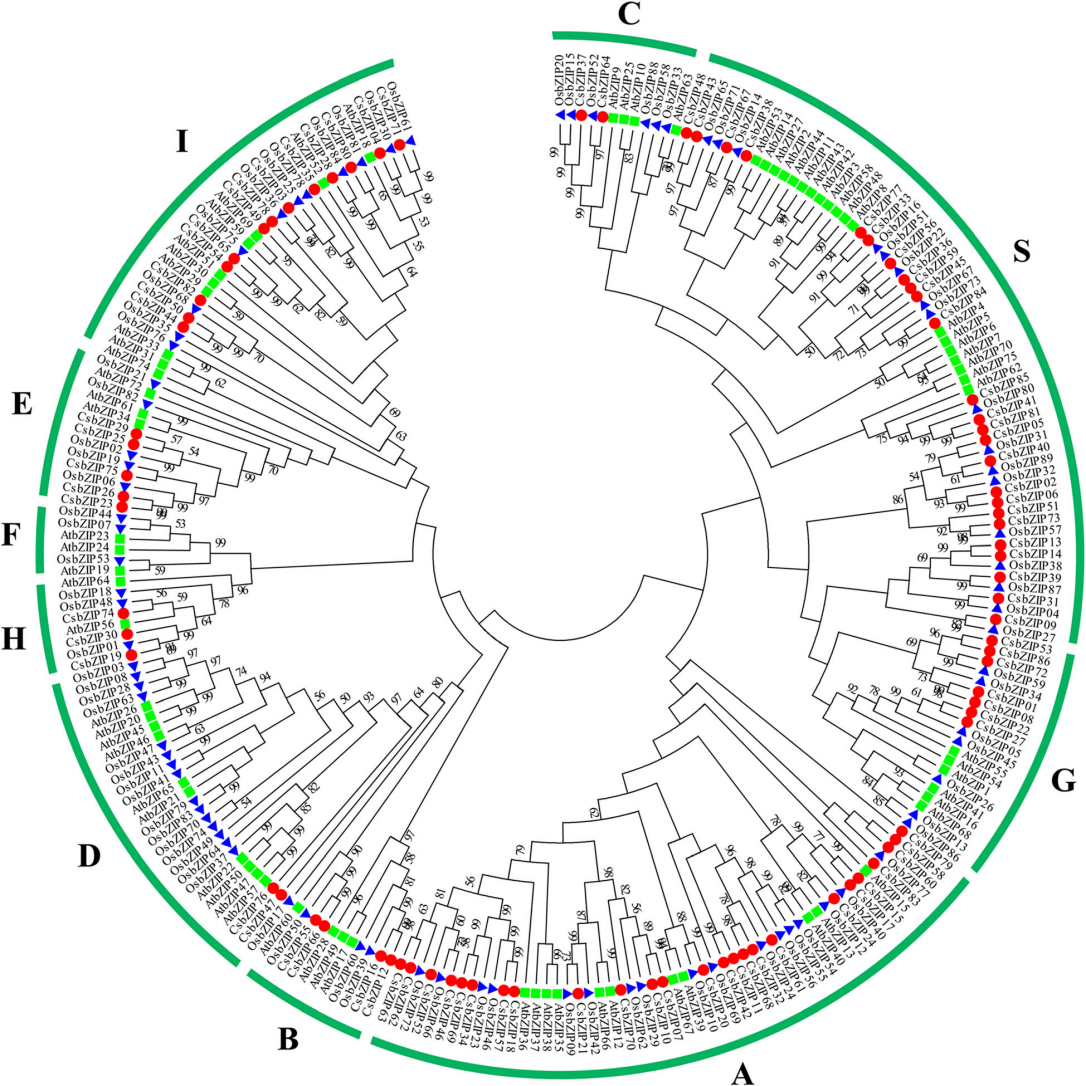
Phylogenetic tree of bZIP proteins in three species, *C. songorica* (red circle), rice (blue triangle), and *Arabidopsis* (green square). The tree was generated with MEGA7 software using the neighbour-joining method

To determine the evolutionary relationship of the CsbZIP proteins and other known bZIP proteins, an unrooted neighbour-joining tree was created with 250 bZIP proteins from three plant species (86 from *C. songorica*, 75 from *Arabidopsis*, and 89 from rice). The results showed that 250 bZIP proteins were grouped into 10 subfamilies, named subfamilies A to I and S. Subfamily A and subfamily S both contained 25 CsbZIP protein members, whereas no CsbZIP proteins were found in subfamily F (Fig. 1). Generally, the CsbZIP proteins had closer relationships with the bZIPs from rice than those from *Arabidopsis*, which is confirmed by the current understanding of plant evolutionary history. To further understand the structural evolution of the *CsbZIP* genes, we analysed the gene structure of the *CsbZIP* genes. Twenty-one *CsbZIP* genes (24.4%) had one exon, 19 of which were clustered into subfamily A. *CsbZIP79*, *CsbZIP85* and *CsbZIP22* contained 11 exons, which was the highest number of exons among the *CsbZIP* genes. In subfamilies A, B, E, H, and I, most *CsbZIP* genes contained < 5 exons, and most of the *CsbZIP* genes in subfamilies C, D, and G contained 6–11 exons (Additional file 2: Figure S1). To obtain insight into the diversity of motifs and functional prediction of the CsbZIP proteins, 20 conserved motifs were identified and designated using the MEME web server. Strikingly, all CsbZIP proteins contained the basic leucine zipper domain motif 1. In addition, motifs 9, 13 and 15 were specific for subfamily I, motif 20 was only found in subfamily H and B, motifs 3, 4 (abscisic acid-insensitive), 5 and 6 only appeared in subfamily A, and motif 12 was present exclusively in subfamily G (Additional file 2: Figure S1 and Additional file 3: Figure S2). In summary, the *CsbZIP* genes have conserved structural and exon-intron organization similarities in the same subfamily.

### Genome synteny and variation analysis of the bZIP family in *C. songorica*

**Fig. 2:**
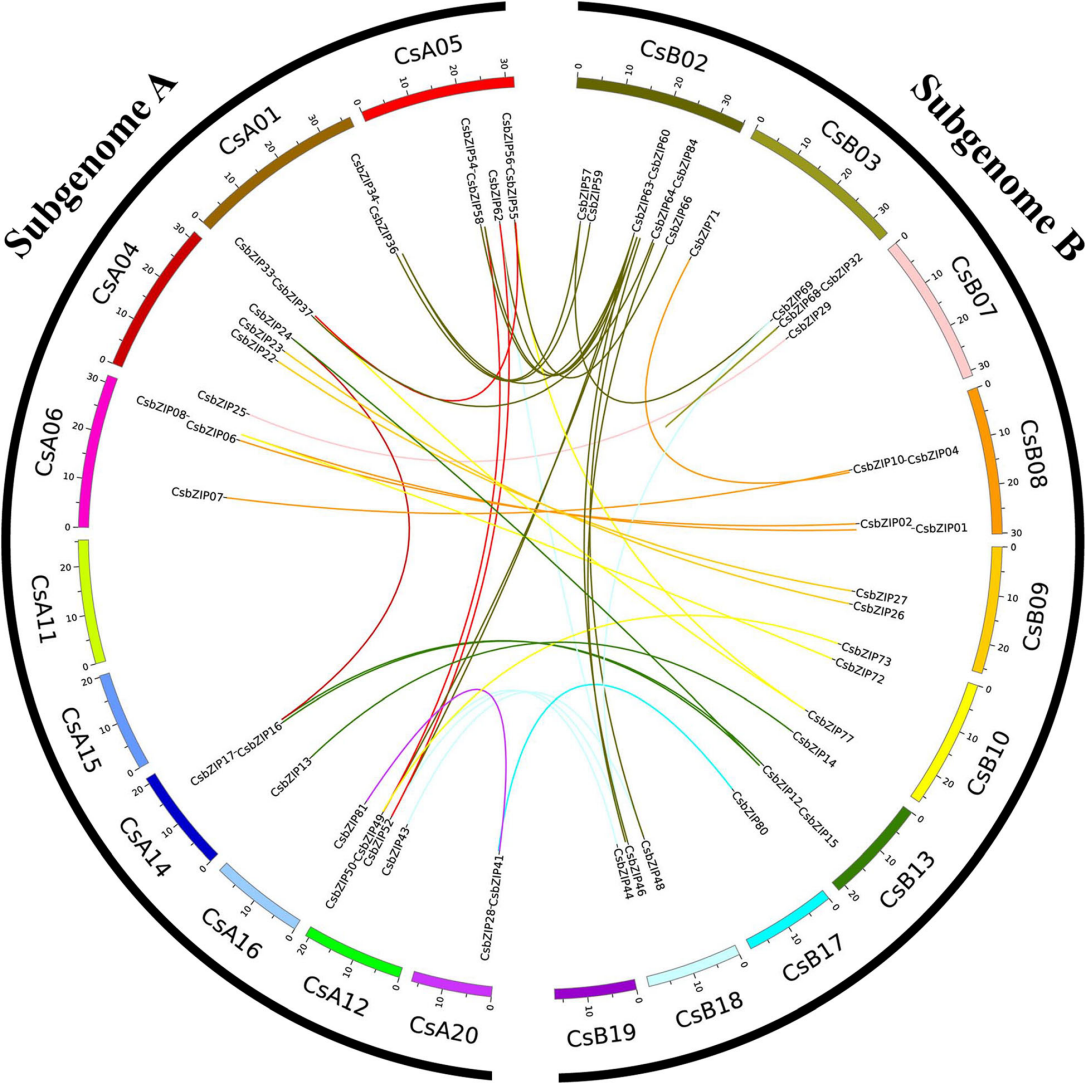
Distribution and synteny analysis of *C. songorica bZIP* genes. In the figure, the 20 *C. songorica* chromosomes are shown in different coloured partial circles, and chromosome numbers are indicated at the top of each bar. Coloured bars indicate *bZIP* syntenic regions in the *C. songorica bZIP* gene family

For gene loci analysis, 82 *CsbZIP* genes (95.34%) were located on 20 *C. songorica* chromosomes, with chromosome CsA11 containing the most (12.2%), followed by chromosomes CsA04, CsB08 and CsA02 with approximately 8.5%, but no *CsbZIP* genes were located on chromosome CsB19 (Additional file 4: Figure S3). Subgenome A and subgenome B of *C. songorica* contained 44 (53.7%) and 38 (46.3%) *CsbZIP* genes, respectively. The average amino acid number and pIs of the CsbZIP proteins in subgenome A were higher than those of the CsbZIP proteins in subgenome B (Additional file 4: Figure S3). In this study, 40 putative paralogous gene pairs were identified in the *C. songorica* genome, including 39 paralogous gene pairs produced by segmental duplication, and 1 paralogous gene pair produced by tandem duplication events with the same chromosomes (*CsbZIP32* and *CsbZIP68*). In addition, 29 paralogous gene pairs were identified from subgenome A to subgenome B of *C. songorica* (Fig. 2 and Additional file 5: Table S2). For example, *CsbZIP14* and *CsbZIP15* were located on CsB13, *CsbZIP13* and *CsbZIP17* on CsA14, whose gene structure and conserved motif were highly similar. For further evolutionary studies of the bZIP family, we calculated the Ka, Ks and Ka/Ks values of paralogous gene pairs based on synteny analysis. For the paralogous gene pairs in *C. songorica*, the frequency distributions of the relative Ks for the paralogous gene pairs peaked at 0–0.4 in *C. songorica* (Fig. 4 and Additional file 5: Table S2).

### Evolutionary and phylogenetic relationship of *C. songorica* and rice bZIP TFs

**Fig. 3:**
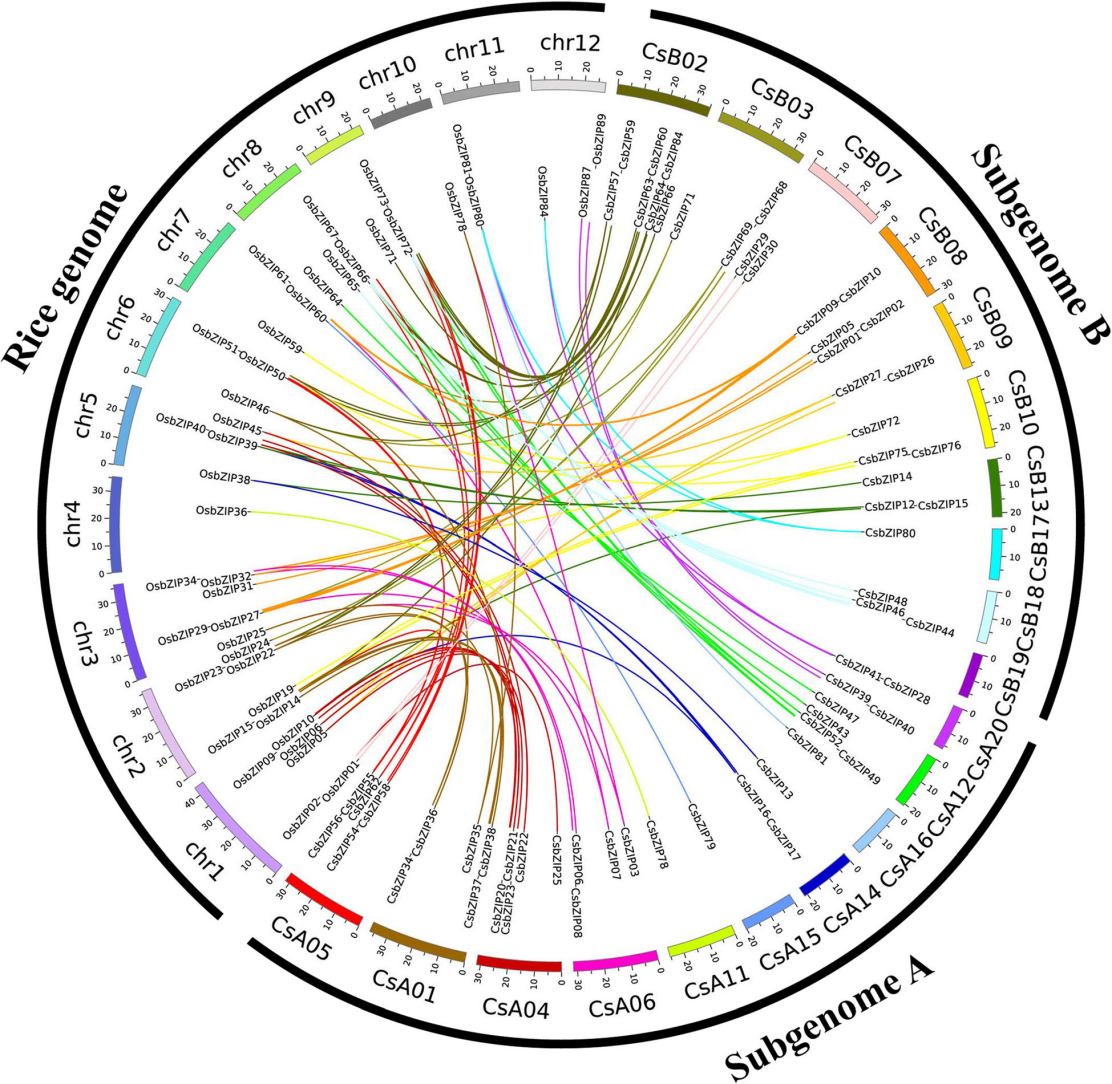
Distribution and synteny analysis of *bZIP* genes between *C. songorica* and rice. In the figure, the 20 *C. songorica* chromosomes are shown in different coloured partial circles, and chromosome numbers are indicated at the top of each bar. Coloured bars indicate *bZIP* syntenic regions in the *C. songorica* and rice *bZIP* gene families. Chr1 to Chr12 belong to rice, whereas subgenome A and subgenome B represent 20 chromosomes of *C. songorica*

**Fig. 4:**
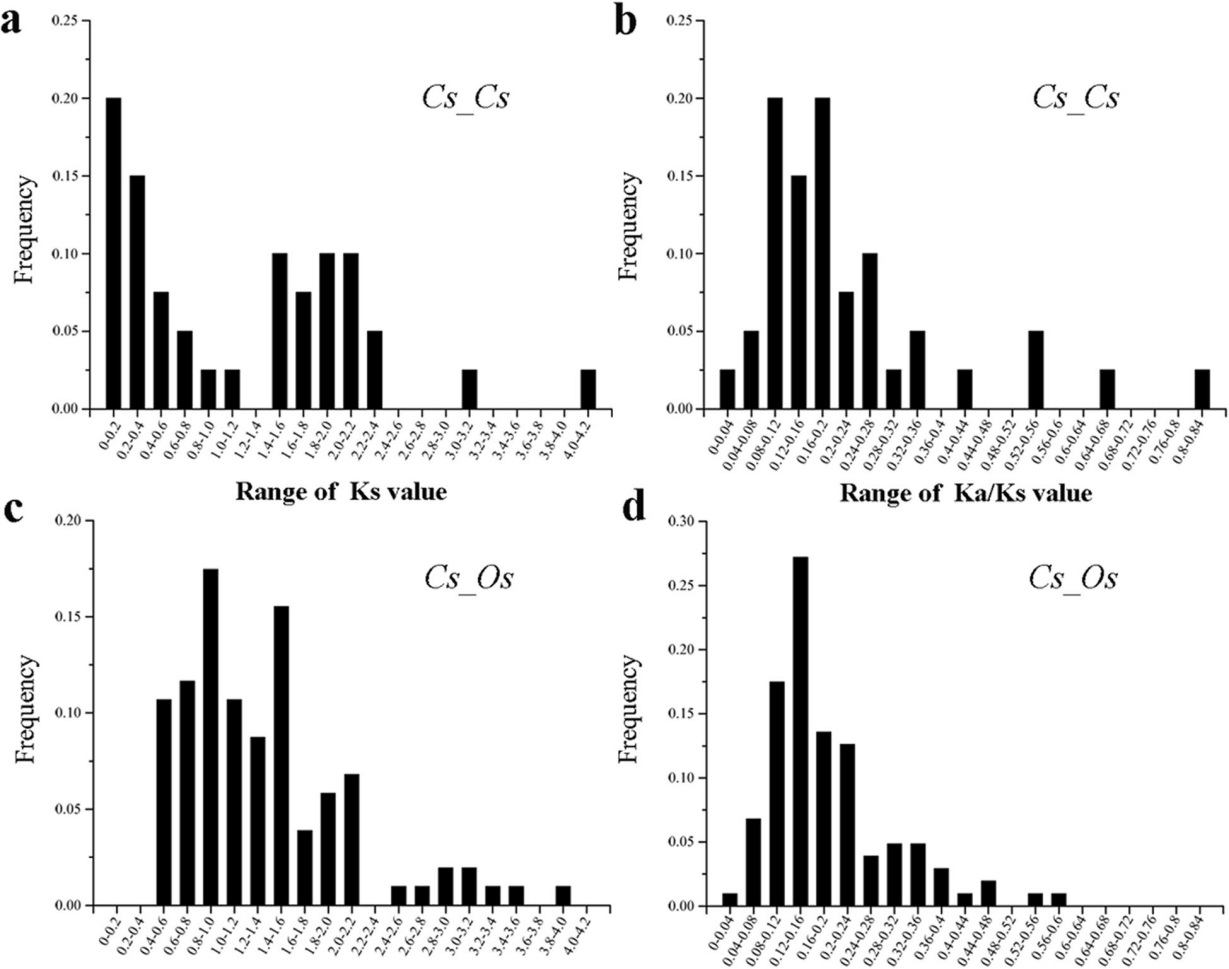
Ks and Ka/Ks value distributions of the *bZIP* genes in the genomes of *C. songorica* paralogous gene-pairs (*Cs-Cs*) and orthologous gene-pairs (*Cs-Os*) between *C. songorica* and rice, depicted with the frequency distribution of relative Ks and Ka/Ks modes. The distribution of Ks and Ka/Ks values were obtained from paralogous gene pairs in the *C. songorica* genome (**a** and **b**) and orthologous gene pairs between *C. songorica* and rice (**c** and **d**)

We further performed a comparative bZIP synteny map of the rice and *C. songorica* genomes. Fifty-four rice *bZIP* genes and 72 *CsbZIPs* were identified as orthologous by large-scale syntenies (Fig. 3 and Additional file 6: Table S3). Among them, we found 12 pairs of syntenic orthologous genes (one-to-one), including *CsbZIP79*-*OsbZIP60*, *CsbZIP78-OsbZIP36*, *CsbZIP67*-*OsbZIP71* and *CsbZIP05-OsbZIP31*. The results suggested that these genes were derived from the same ancestor of rice and *C. songorica*. The relationship of one *CsbZIP* gene corresponding to multiple *OsbZIP* genes was also found, such as *CsbZIP84*-*OsbZIP67*/*OsbZIP73* and *CsbZIP17*-*OsbZIP12*/*OsbZIP40*. Additionally, syntenic orthologous gene pairs of one *OsbZIP* corresponded to multiple *CsbZIP* genes. For instance, *OsbZIP77*-*CsbZIP58*/*CsbZIP60*, *OsbZIP78*-*CsbZIP03*/*CsbZIP35*, and *OsbZIP72*-*CsbZIP46*/*CsbZIP52*/*CsbZIP62*/*CsbZIP63*. Strikingly, syntenic orthologous gene pairs with four *CsbZIP* genes corresponded to the same two genes: *CsbZIP63*/*CsbZIP62*/*CsbZIP52*/*CsbZIP46*-*OsbZIP72*/*OsbZIP66* (Fig. 3 and Additional file 6: Table S3). For the duplicated orthologous gene pairs, the Ks peaked at 0.4–1.6 between *C. songorica* and rice (Fig. 4 and Additional file 6: Table S3). The Ka/Ks ratios peaked between 0.08–0.2 for the paralogous gene pairs, and the Ka/Ks ratios between the rice and *C. songorica* genomes were distributed at 0.08–0.24, with the highest in the *CsbZIP01*-*OsbZIP34* pair (Ka/Ks = 0.58).

### Identification of *cis-*elements in the *CsbZIP* promoters and LTR retrotransposon insertions in the *CsbZIP* introns

**Fig. 5:**
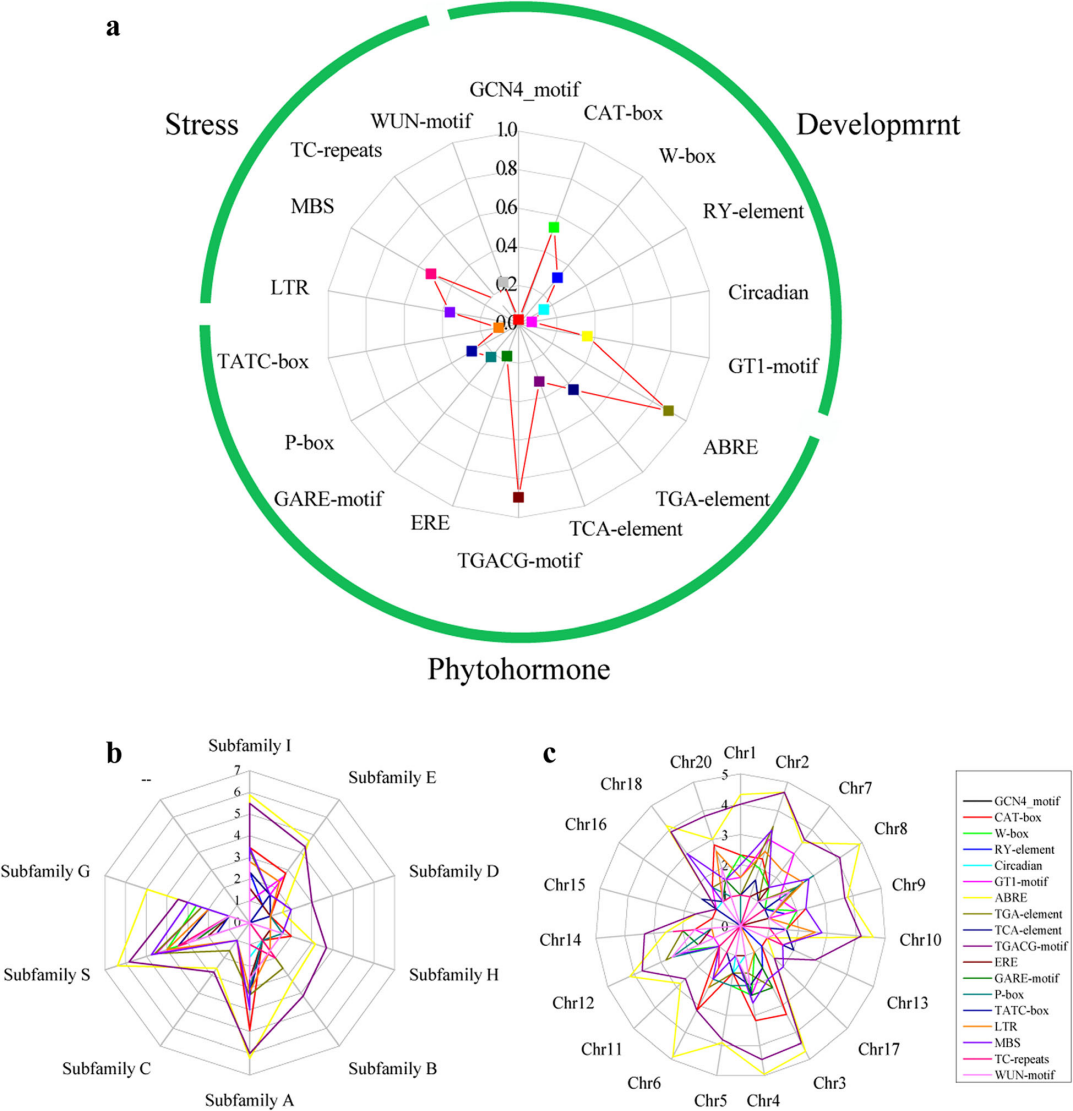
Statistical analysis for *cis*-elements of *CsbZIP* genes. **a** Proportions of genes with various *cis*-elements among the *CsbZIP* genes. **b** Subfamily distributions (log_10_(*cis*-element number + 1). **c** Chromosome distributions (log_10_(*cis*-element number + 1))

To explore the mechanisms of the *CsbZIP* genes in the stress response and developmental process, we searched for 18 *cis-*elements in the promoter region of the *CsbZIP* genes, which were predicted to be involved in development, abiotic stress and phytohormone response. The six most common *cis-*elements included the TGACG-motif (89.5%), abscisic acid responsive element (ABRE, 89.5%), CAT-box elements (53.4%), drought stress element (MBS, 52.3%) and LTR (low-temperature responsiveness, 36%) in the *CsbZIP* promoters (Fig. 5a and Additional file 6: Table S3). The CGTCA-motif and TGACG-motif are involved in MeJA stress, while ABREs are involved in ABA stress. The CAT-box elements and MBS are involved in development and drought stress, respectively. Additionally, 30 *CsbZIP* genes contained the salicylic acid responsive elements (TCA-element), which were involved in the salicylic acid response. Notably, the promoters of 13 *CsbZIP* genes (15.1%) contained at least six *cis-*elements (Additional file 7: Table S4). The analysis of chromosome distributions indicated that different *cis-*elements preferred some chromosomes. For distance, TGA-elements were enriched in CsB02 and CsA12. Likewise, some *cis-*elements were distributed in rare chromosomes, such as GCN4_motif (Fig. 5c and Additional file 6: Table S3). Subfamily G contained all identified *cis-*elements in the promoters of the *CsbZIP* genes. Compared with other families, subfamily S had the most LTRs, MBSs, ABREs and TC-repeats (Fig. 5b and Additional file 7: Table S4).

**Fig. 6:**
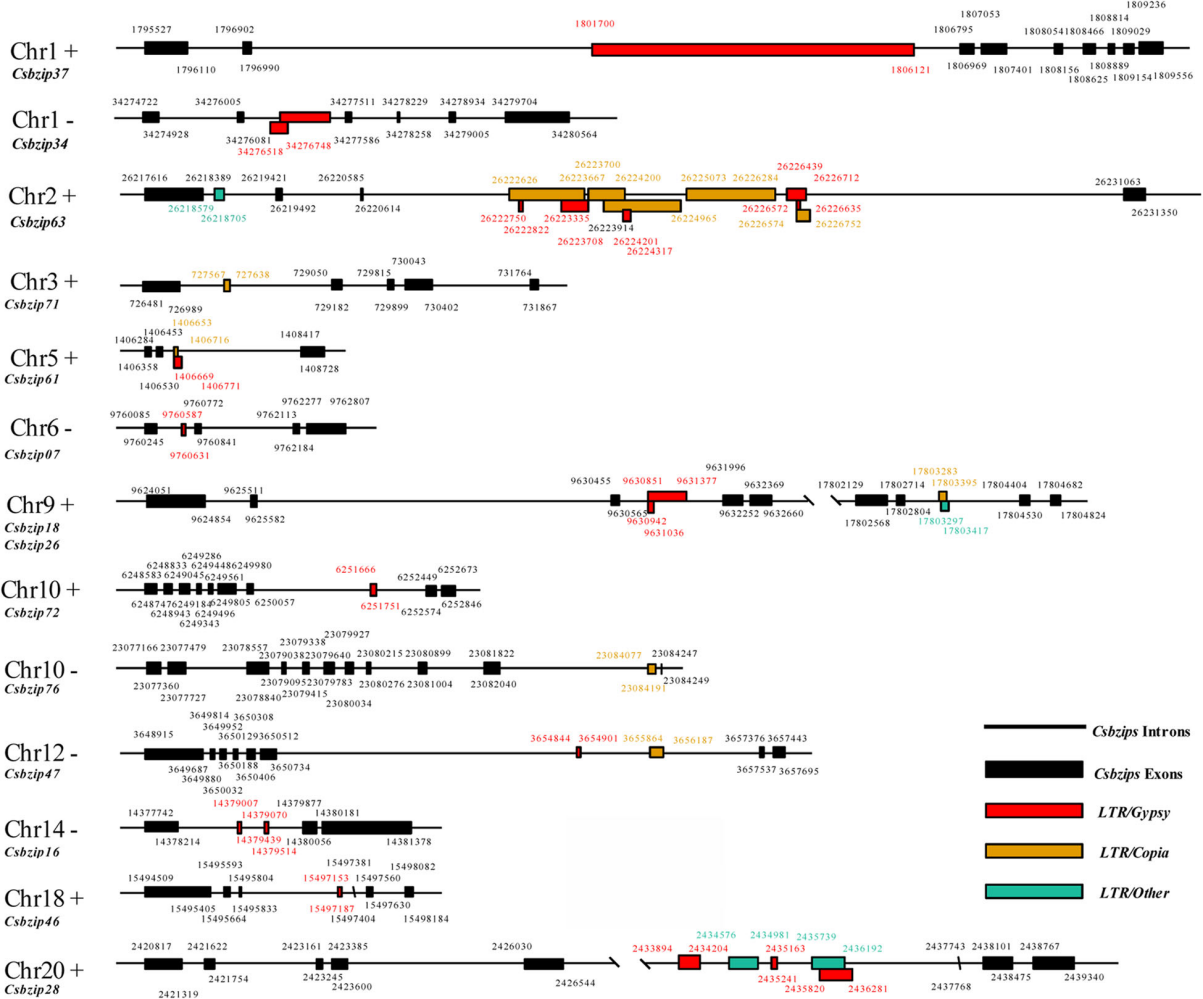
Insertion of the LTR retrotransposon into the *CsbZIP* intron. The black boxes and lines indicate the *CsbZIP* exons and introns, respectively. The red boxes and orange boxes indicate *gypsy* and *copia* elements of LTR retrotransposon, respectively. The green boxes indicate other LTR retrotransposon families

Insertion of the LTR retrotransposon may affect gene structure and gene expression. Here, 14 *CsbZIP* genes (16.3%) were identified for the insertion of the LTR retrotransposon in the intron region (Fig. 6). *CsbZIP63* contained the most LTR retrotransposon elements, including 5 *copia* elements, 4 *gypsy* elements and 1 other element. *CsbZIP46*, *CsbZIP72*, *CsbZIP07* and *CsbZIP37* only contained one *gypsy* element. In contrast, *CsbZIP76* and *CsbZIP71* only contained one *copia* element. Furthermore, 4 *CsbZIP* genes contained both *copia* and *gypsy* elements (Fig. 6).

### Identification of dimorphic floret-associated bZIP family members

**Fig. 7:**
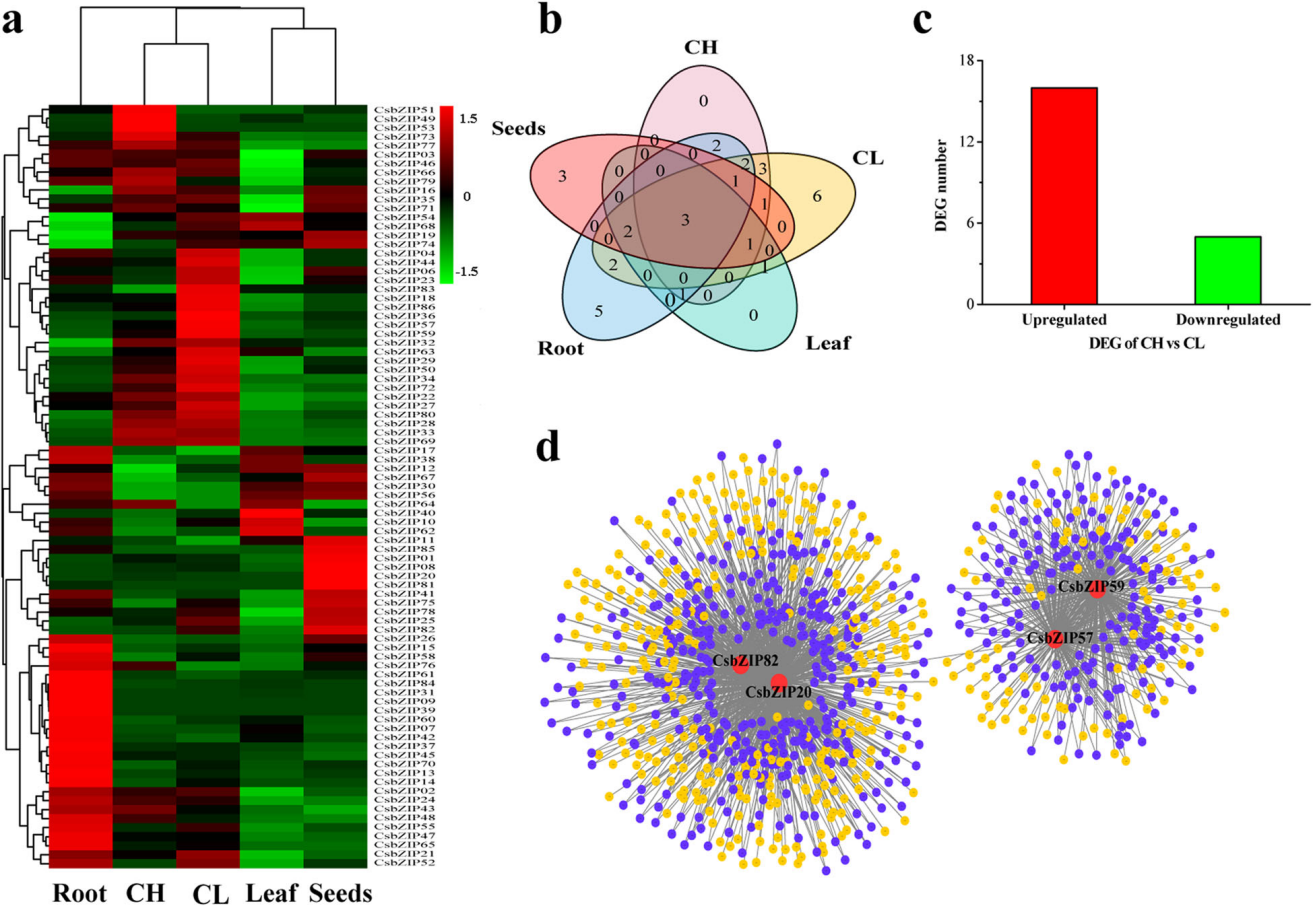
Expression and co-expression network analysis of the *CsbZIP* genes in CH and CL flowers. **a** Heat map of all *CsbZIP* genes in different tissues based on transcriptome datasets. The expression values (FPKM) were normalized. **b** Venn diagram showing overlap of highly expressed *CsbZIP* genes (FPKM ≥20) in different tissues. **c** Number of differentially expressed *CsbZIP* genes between CH and CL flowers. **d** Co-expression network of *CsbZIP* genes with DEGs between CH and CL flowers. The grey lines between two nodes indicate co-expression relationships. The purple solid circles represent overlapping genes

To study the expression pattern of the *CsbZIP* genes in different organs, we performed transcriptome analysis of *C. songorica* tissues, including the chasmogamous (CH) flowers, cleistogamous (CL) flowers, seeds, leaves, and roots (Fig. 7a). Eighty-one (94.2%) *CsbZIP* genes expressed at least in one tissue (FPKM≥1), and 40 (46.5%) genes were expressed in all tissues (FPKM≥1; Additional file 8: Table S5). Furthermore, 33 genes showed high expression levels in at least one tissue (FPKM≥20). Three genes were highly expressed in all tissues, including *CsbZIP10*, *CsbZIP13* and *CsbZIP38* (Fig. 7b). Strikingly, nine genes were only highly expressed in flowers but not in roots and leaves tissues; five genes showed high expression levels only in roots but not in flower tissues (Fig. 7b).

*C. songorica* can produce dimorphic floret (chasmogamous, cleistogamous) on the same individual plant that appear in different positions. We identified 21 differentially expressed |log_2_(fold change) ≥ 1| *CsbZIP* genes between CH and CL flowers, including five downregulated and 16 upregulated *CsbZIP* (Fig. 7c). Gene ontology (GO) annotation showed that these genes were involved in the regulation of biological process (GO:0050789), developmental maturation (GO:0021700), gametophyte development (GO:0048229), response to stress (GO:0006950), pollen development and maturation (GO:0009555 and GO:0010152). Strikingly, *CsbZIP58* and *CsbZIP60* were identified as *FD* (*FLOWERING LOCUS D*) gene, which participated in floral induction. *CsbZIP60* participated in pollen maturation (GO:0010152) and pollen development (GO:0009555) (Additional file 9: Table S6).

Four dimorphic florets related *CsbZIP* genes (*CsbZIP20*, *CsbZIP57*, *CsbZIP59* and *CsbZIP82*) were selected for co-expression analysis (Fig. 7d and Additional file 10: Table S7). Nine hundred and sixty co-expressed genes were identified and some showed overlap with these four genes. Gene ontology analysis indicated that these genes were involved in organ development (GO:0048513), flower development (GO:0009908), floral organ development (GO:0048437), reproductive structure development (GO:0048608) and response to stress (GO:0006950; Additional file 10: Table S7 and Additional file 11: Figure S4). This result suggested that the *CsbZIP* genes may participate in dimorphic florets development by regulating floral organ development and flower development.

### Identification of abiotic stress-related *CsbZIP* genes

**Fig. 8:**
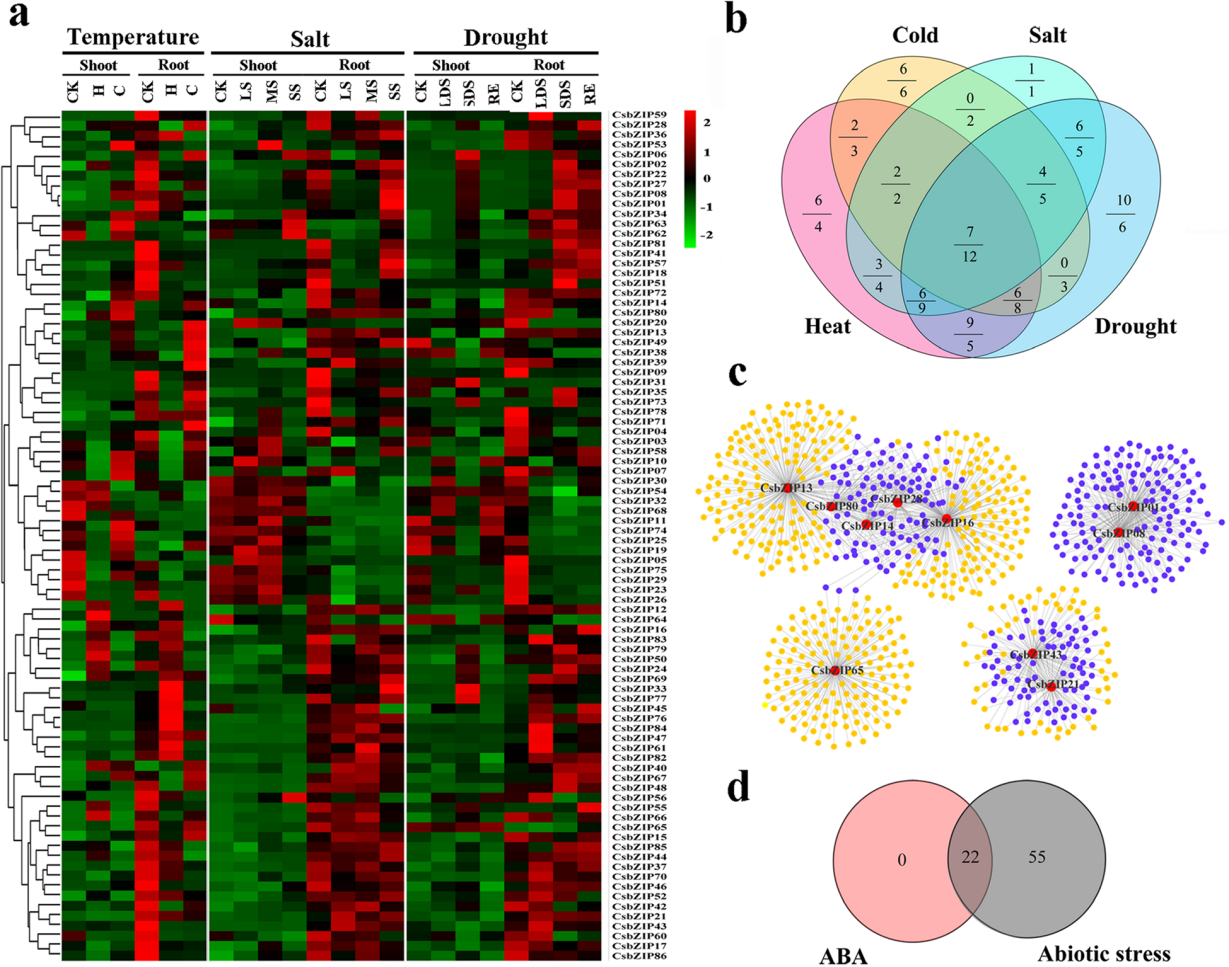
Expression and co-expression network analysis of *CsbZIP* genes under abiotic stress. **a** Heat map of all *CsbZIP* genes under different abiotic stresses based on transcriptome datasets. The expression values (FPKM) were normalized. **b** Venn diagram showing the overlap of differentially expressed *CsbZIP* genes under abiotic stress. The letters on the line and below the line indicate the differentially expressed *CsbZIP* gene numbers in the shoots and roots, respectively. **c** Co-expression network of the *CsbZIP* genes with DEGs under abiotic stress. The grey lines between two nodes indicate co-expression relationships. The purple solid circles represent overlapping genes. **d** Venn diagram showing the overlap of differentially expressed *CsbZIP* genes under abiotic stress and ABA stress. *H*: heat stress; *C*: cold stress; *LS*: light salt stress; *MS*: moderate salt stress; *SS*: severe salt stress; *LDS*: light drought stress; *SDS*: severe drought stress; *RE*: recovery

**Fig. 9:**
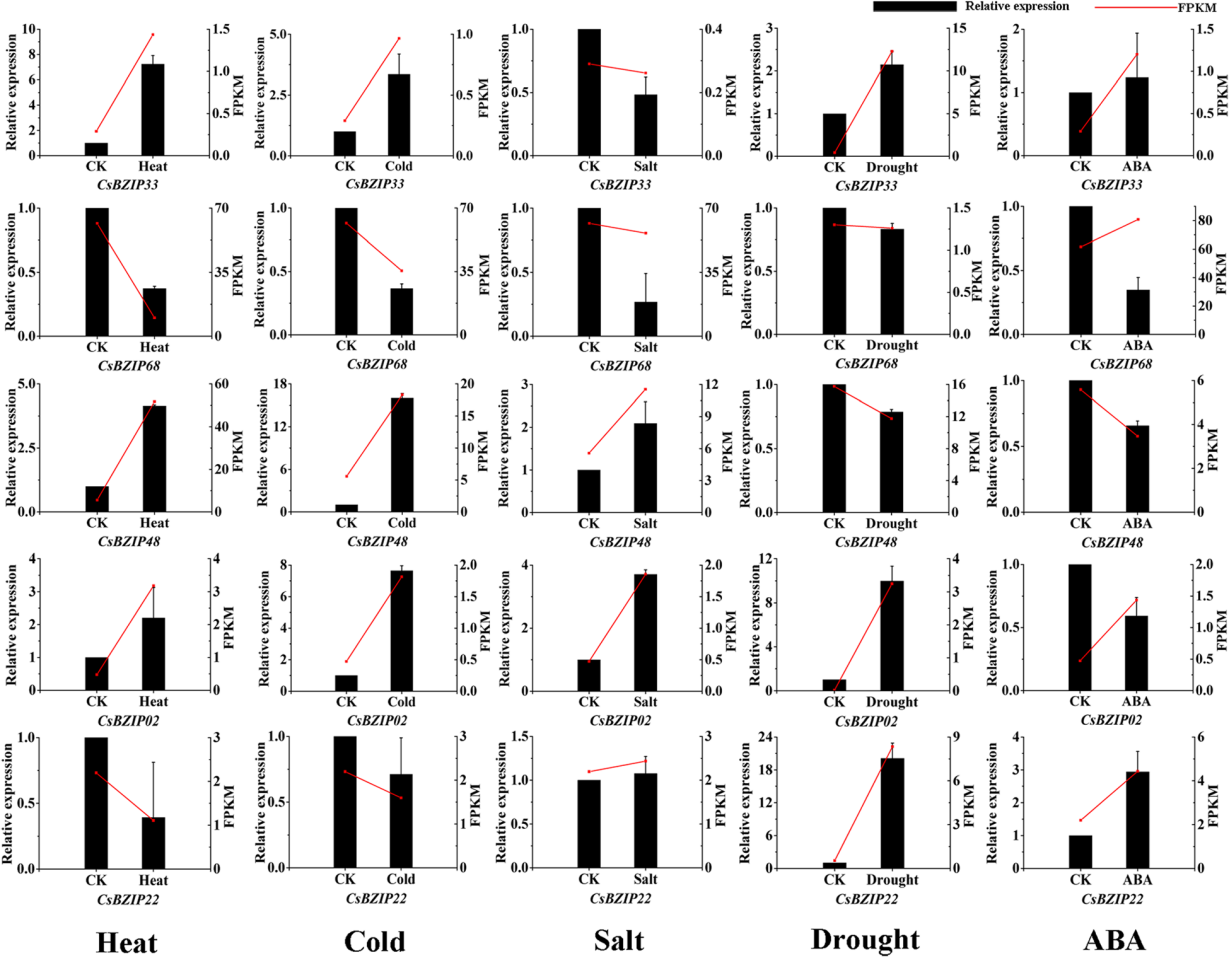
Confirmation of the expression patterns of the *CsbZIP* genes under abiotic stress in shoots using quantitative qRT-PCR. The values shown are the means ± standard deviation of three replicates. *CsGAPDH* was used as the reference gene

We used the transcriptome data sets of *C. songorica* treated with high temperature (heat), low temperature (cold), salt and drought stress to determine the impact of the *CsbZIP* genes on abiotic stress. Seventy-four (87.2%) and seventy-eight (90.7%) *CsbZIP* genes showed gene expression at least in one stress condition (FPKM ≥1) in shoots and roots (Fig. 8a and Additional file 12: Table S8), respectively. Furthermore, 68 (79.1%) and 75 (87.2%) *CsbZIP* genes were differentially expressed |log_2_(fold change) ≥ 1| under at least one treatment in shoots and roots, respectively (Fig. 8b and Additional file 12: Table S8). As shown in Fig. 9b, 41, 27, 29 and 48 differentially expressed *CsbZIP* genes were identified in *C. songorica* shoots under heat, cold, salt and drought stress, respectively. Compared to the controls, there were 47, 41, 40 and 53 differentially expressed *CsbZIP* genes in roots under the heat, salt, cold and drought stress treatments, respectively (Fig. 8b and Additional file 12: Table S8). Interestingly, seven and 12 of these genes overlapped under four abiotic stress conditions in shoots and roots, respectively. A total of 77 *CsbZIP* genes were differentially expressed in both shoots and roots under four abiotic stress conditions. These genes were mainly distributed in subfamilies A, E and S. In subfamily E, four out of five genes were differentially expressed in shoots under abiotic stress. The heat and drought treatments had the most overlapping genes (34 in shoots; 28 in roots), whereas the cold and salt stress treatments had the fewest overlapping genes (13 in shoots, 21 in roots; Fig. 8b and Additional file 12: Table S8).

Ten stress-related *CsbZIP* genes were found in the co-expressed network analysis. As shown in Fig. 7c, 835 co-expressed genes were found, and some showed overlap with these genes. Furthermore, we analysed the GO annotation of these co-expressed genes, and some major stress-related GO terms were found response to stimulus (GO:0050896), response to hormone (GO:0009725), and metabolic process (GO:0008152). Strikingly, we also found that some genes were involved in flower development, including reproductive process (GO:0022414), reproductive system development (GO:0061458), reproductive structure development (GO:0048608), flower development (GO:0009908), floral organ development (GO:0048437), and pollen development (GO:0009555; Additional file 13: Table S9). We further identified the ABA-dependent and ABA-independent abiotic stress-responsive *CsbZIP* genes. A total of 22 *CsbZIP* genes were differentially expressed in the ABA treatment. Among these genes, eight *CsbZIP* genes were classed to subfamily A. Furthermore, 22 *CsbZIP* genes were also responsive to both the ABA and abiotic stress treatments in *C. songorica*, while 55 *CsbZIP* genes were specifically responsive to abiotic stress, indicating that more ABA-independent *CsbZIP* genes were involved in response to abiotic stress.

The expression patterns of some related *CsbZIP* genes were verified by RT-qPCR under salt stress (100 mM NaCl; 24 h), drought stress (2% soil water content), heat stress (40 °C; 24 h), cold stress (4 °C; 24 h) and ABA stress (100 μM; 24 h). As shown in Fig. 9, the result was relatively consistent with similar trends, indicating that the selected *CsbZIP* genes were significantly induced by these five stresses. For example, *CsbZIP48*, *CsbZIP02* and *CsbZIP33* were upregulated under heat and cold stresses. *CsbZIP02* and *CsbZIP22* were upregulated under drought stress.

## Discussion

*C. songorica* is an allotetraploid plant and a major xerophyte perennial desert plant native to Northwest China that provides valuable genetic resources for understanding and improving stress resistance in plants. Currently, we have completed whole-genome sequencing and transcriptome sequencing of *C. songorica*. Many studies have shown that the bZIP family participates in different biological processes, including plant development, flowering and response to environmental stresses [35]. Although the functions of bZIP TFs are diverse, a genome survey of bZIP family genes has not yet been reported in *C. songorica*. Here, 86 bZIP family genes were found in the *C. songorica* genome. The distribution of the *CsbZIP* genes was not different in the subgenome of *C. songorica* (Additional file 2: Figure S1). Compared to other plants, the result suggested that the bZIP family did not exhibit significant expansion in *C. songorica*. For example, 75 bZIP TFs were identified in *Arabidopsis* [9], 77 were found in cassava [5], and 64 were recognized in cucumber [36]. Evolutionary analysis showed that bZIP TFs from three species could be classified into 10 subfamilies by multiple sequence alignments. Strikingly, no *CsbZIP* genes were distributed in subfamily F, which is involved in Zn transport (Fig. 1). In *Arabidopsis*, *AtbZIP19*, *AtbZIP23* and *AtbZIP24* from subfamily F were suggested to improve the plant resistance under Zn-limiting treatments [37]. These results suggested that *C. songorica* lost these functional genes during evolution.

Gene structure analysis indicated that the *CsbZIP* genes introns presented a large difference with numbers ranging from 0 to 10, but there was a similar gene structure in each subfamily (Additional file 2: Figure S1). We found that approximately 24% of *CsbZIP* genes only had an exon and were mainly clustered in subfamily S, with the same results as soybean and banana [21, 38]. In addition, duplicated gene pairs were also found to be classified in the same subfamily by phylogenetic analysis. For example, the segmental pairs *CsbZIP32*-*CsbZIP68* (3 exons) and *CsbZIP25*-*CsbZIP29* (4 exons) were classified into subfamilies A and E, respectively. The *CsbZIP* genes of subfamilies D, C and G had more exons than all other subfamilies (Additional file 2: Figure S1). This result is supported by results from studies of soybean and *Brassica napus* [27, 38]. Research has shown that the rate of intron gain by segmental duplication is slower than the rate of intron loss in rice [39]. These results might indicate that the original genes were distributed in subfamilies D, C and G. The mechanism of exon/intron gain/loss caused diversification for the gene family [40]. Conserved motif analysis showed that all the CsbZIP proteins contained the typical bZIP domain, Furthermore, we found that motif 4 (abscisic acid-insensitive) was involved in ABA, and motif 5 was specific to subfamily A (Additional file 2: Figure S1). The DNA-binding ability of bZIP transcription factors is depend on the basic region of the bZIP domain. Then, the specific DNA-binding is controlled by certain key amino acid residues present from the basic and hinge regions of bZIP domain. Plant bZIP transcription factors can bind to some motif ACGT core DNA sequences, including G-box, ABRE, GCN4_motif, etc. [9]. In this study, some *cis*-acting elements also were identified in the upstream of *CsbZIP* genes. In *Arabidopsis*, some *AtbZIP* genes can regulate transcription of ABA dependent genes that interacting with ABRE (Abscisic acid responsive element) *cis*-element on promoters, including *ABF1*, *ABF2*, *ABF4* [41]. They belong to subfamily A and abscisic acid-responsive element-binding factors (ABF) [7]. In *C. songorica*, 84% *CsbZIP* genes contained at least one ABRE *cis*-element on promoters and showed the expression change under ABA treatment in subfamily A. Furthermore, *AtbZIP11* have been confirmed to modulate transcription of target gene that carry the G-box *cis*-element [42]. In this study, *CsbZIP72* and *CsbZIP76* contained the GCN_motif *cis*-element (G-box) that is the recognition site for bZIP transcription factors in promoters. In rice, *RISBZ1*, a bZIP transcription factor, are involved in storage of endosperm based on interacting with GCN4_motif [43]. These results indicated that *CsbZIP72* and *CsbZIP76* also participated in storage process. Together, gene structure and conserved motif analysis indicated that the relationship of *CsbZIP* genes in the same subfamily were closer than those in other subfamilies during the gene evolution process.

The duplication event of genes caused the generation of new genes and gene family expansion. The mechanism mainly included segmental duplication, tandem duplication, and transposition events. In this study, almost all paralogous gene pairs from *C. songorica* were distributed between chromosomes. In addition, 29 paralogous gene pairs (72.5%) arose from the duplication of subgenome A and subgenome B of *C. songorica* (Fig. 2). In this whole-genome analysis, we also found that *C. songorica* had undergone a whole-genome duplication event in evolutionary history. These results suggested that the expansion of the *C. songorica* gene family was produced by whole-genome duplication events. The mechanism of comparative genomics divided the syntenic blocks by genome structures. The synteny analysis can identify the functional and evolutionary relationship between different species. We performed syntenic and phylogenetic comparisons between rice and *C. songorica* and identified orthologous gene pairs. A total of 52 *OsbZIP* and 73 *CsbZIP* genes (84.9%) were identified as orthologs. We found 12 single orthologous gene pairs (11.7%) between *C. songorica* and rice, indicating that these genes might be contained in the genome of the common ancestor of the two species (Fig. 3). Most gene pairs (88.3%) showed a substantially complex relationship, including single *CsbZIP* genes corresponding to the multiple *OsbZIP* genes or single *OsbZIP* genes corresponding to the multiple *CsbZIP* genes. Furthermore, we further calculated the Ks values and Ka/Ks ratios for the orthologous (*Cs-Os*) and paralogous (*Cs-Cs*) gene pairs. When Ka/Ks < 1, this indicates that the gene has purifying selection, but Ka/Ks ≥ 1 indicates positive selection for the gene [44, 45]. The Ka/Ks ratios of the orthologous gene pairs were lower than 0.6, indicating they might have progressed through a purifying selection during the long evolutionary history between the rice and *C. songorica* genomes (Fig. 4).

Extensive studies have indicated the important functions of the bZIP transcription factors in regulating various stress signalling pathways. In *Arabidopsis*, overexpressing *TabZIP6* from wheat (*Triticum aestivum* L.) decreased the freezing tolerance of transgenic *Arabidopsis* seedlings [46]. Overexpression of *GhABF2*, a *bZIP* gene from cotton (*Gossypium hirsutum* L.), significantly improved drought and salt stress tolerance both in transgenic *Arabidopsis* and cotton plants [47]. Although some stress-related *bZIPs* have been identified in plants, research on *C. songorica* is still lacking. Here, 79.1 and 87.2% abiotic stress-related *CsbZIP* genes were identified in shoots and roots, respectively. In addition, 7 and 12 genes showed differential expression under four stress conditions (Fig. 8b). These results indicated that *CsbZIP* genes are involved in the regulation of abiotic stresses. Gene expression analysis demonstrated that there were more stress-responsive *CsbZIP* genes for drought and heat than for cold and salt stress-responsive *CsbZIP* genes, suggesting that *CsbZIP* genes might have important biological functions in response to drought and high temperature. This result is supported by the fact that *C. songorica* is a native grass in desert grasslands. Furthermore, all ABA-responsive *CsbZIP* genes were differentially expressed under abiotic stresses. These results indicate that the *CsbZIP* genes participate in ABA-dependent and ABA-independent pathways.

In flowering plants, cleistogamy is a special mating system that has been found in approximately 700 species [48–50], such as *Pseudostellaria heterophylla* and *Viola philippica* [51, 52]. The types of cleistogamy can be divided into dimorphic cleistogamy, complete cleistogamy and induced cleistogamy [50]. Cleistogamy is suspected to be beneficial to plants because CL flowers can ensure seed production by self-pollination under extreme environmental conditions [53]. *C. songorica*, which undergoes dimorphic cleistogamy, produces both closed (cleistogamous) flowers and open (chasmogamous) flowers on the same individual, but the flowers appear in different positions. Unlike CH flower production at the apical meristem, the CL flower is produced in the leaf sheath and plays a crucial role in seed yield in *C. songorica* [30]. In this study, 21 *CsbZIPs* were differentially expressed between CH and CL flowers, including 2 *FD* genes (Fig. 7c). In *Arabidopsis*, *FT* genes were found to regulate the flowering time and floral induction at the meristem [10]. *APETALA1* (*AP1*) is target genes for *FT* genes and belong to the classic ABCE model. *AP1* is involved in floral meristem specification and perianth identity [54, 55]. In *P. heterophylla*, the A class gene *AP1* is highly expressed in CL flowers [51]. Furthermore, *FD*, a bZIP TFs, was found to play a core role in the *FT* activation of floral identity genes such as *AP1* in *Arabidopsis* [10]. In our study, two *FD* genes were differentially expressed between CH and CL flowers in *C. songorica* (*CsbZIP58* and *CsbZIP60*). Strikingly, *CsbZIP58* and *CsbZIP60* were paralogous gene pairs and were orthologous genes for *OsbZIP77,* which is named *OsFD1*. *OsFD1* functions in the activation of the *AP1*/*FUL* genes and promotes flowering [56].

**Fig. 10:**
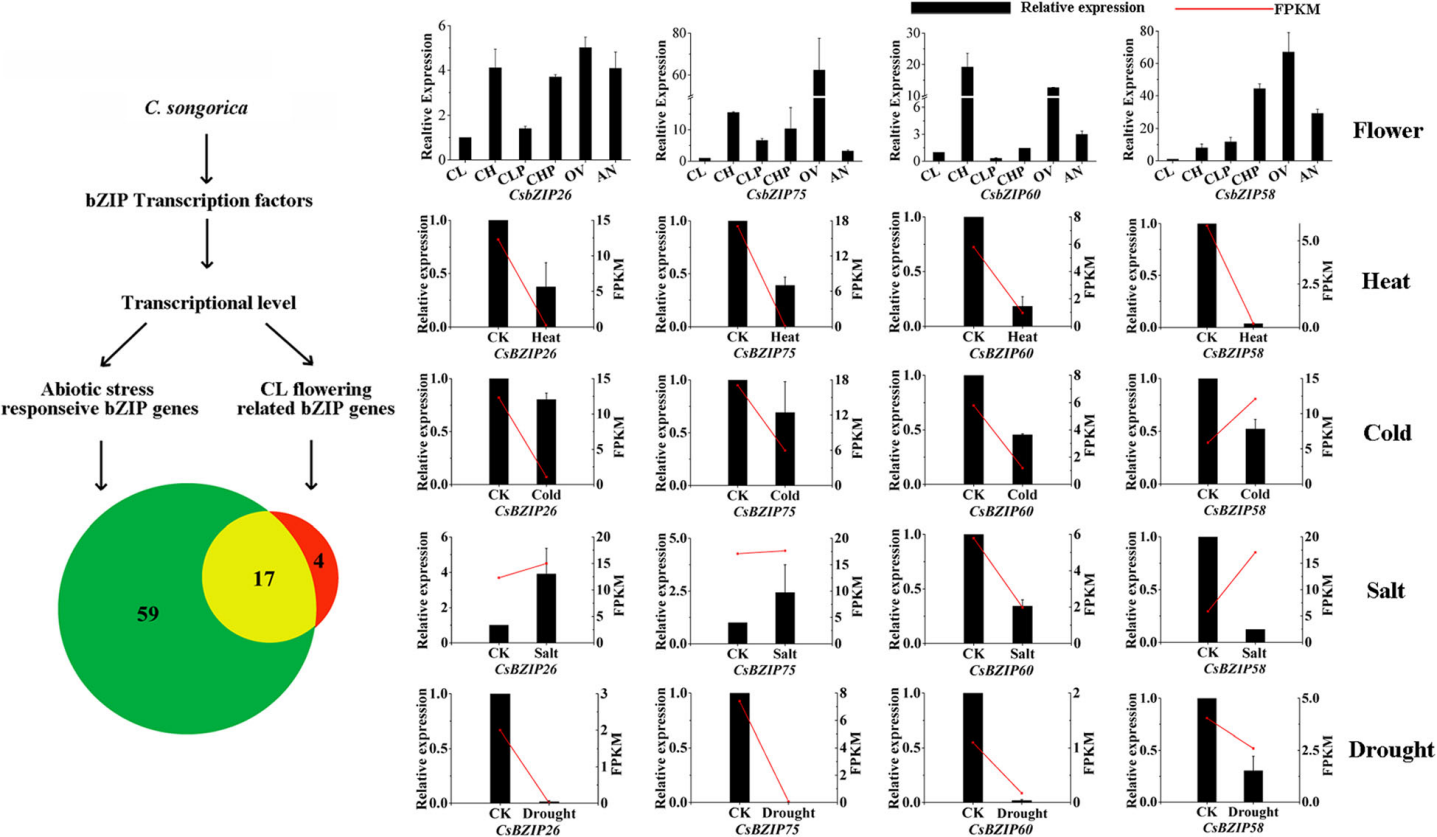
Model of functional divergence of CL flowering-related and stress-responsive *CsbZIP* genes in *C. songorica*. Confirmation of the expression patterns of the *CsbZIP* genes using quantitative qRT-PCR. CH: chasmogamous flowers; CL: cleistogamous flowers; CHP: chasmogamous flower primordium; CLP: cleistogamous flower primordium; OV: chasmogamous ovary; AN: chasmogamous anther. The values shown are the means ± standard deviation of three replicates. *CsGAPDH* was used as the reference gene

The WGCNA analysis showed that some stress-responsive *CsbZIP* genes were co-expressed with genes involved in flower development. Additionally, some CL flower-related *CsbZIP* genes were also co-expressed with genes that responded to stress (Fig. 7d and Fig. 8c). We further found that 17 common *CsbZIP* genes showed differential expression under stress response and during CL flowering, revealing that these genes may involve numerous developmental processes in *C. songorica* (Fig. 10). Four *CsbZIP* genes were selected to verify the expression pattern in CL flowers and CH flowers and under abiotic stress by RT-qPCR. The expression patterns of selected genes showed significant expression changes between the CH and CL flowers and floral primordium (CHP, CLP). Strikingly, these genes also showed significant expression changes and were barely downregulated under abiotic stress. For example, *CsbZIP26*, *CsbZIP75* and *CsbZIP60* were differentially expressed under drought stress. *CsbZIP60* and *CsbZIP58* were differentially expressed under heat stress (Fig. 10). In *Cardamine kokaiensis*, 69 differentially expressed genes related to floral development and cold stress were identified in CH and CL flowers [57]. Furthermore, the study of *P. heterophylla* also indicated that significantly differentially expressed between CH and CL flowers were involved in defence responses [51]. These results may show that CL flowers might be affected by environmental stresses.

## Conclusion

In summary, this study represents the first comprehensive identification and analysis of the bZIP TF family in *C. songorica*. CL flower development and stress response *CsbZIP* genes were highlighted for their expression patterns and expression networks. These results may provide useful insights into the *CsbZIP* genes in the stress response and CL flower development in future studies.

## Methods

### Identification of the *bZIP* genes in *C. songorica*

The bZIP protein and gene sequences from rice (*Oryza sativa*) and *Arabidopsis* were acquired from Phytozome12 (https://phytozome.jgi.doe.gov/pz/portal.html). BLAST searches were used to identify the candidate bZIPs in the *C. songorica* genome (not published data) with rice and *Arabidopsis* bZIP sequences as a query (*e*-value cut-off>1e^− 5^). Then, conserved domain and redundant sequence of all possible protein sequences were further examined the with PFAM (https://pfam.sanger.ac.uk/; *e*-value cut-off>1e^− 5^; PF00170) and CD-HIT (http://weizhongli-lab.org/cdhit_suite/cgi-bin/index.cgi) tools, respectively.

### Protein properties, phylogenetic classification, gene structure and conserved motif analysis

The CsbZIP amino acid residues, grand average of hydropathicity, isoelectric points and molecular weights were analysed by ExPaSy (http://web.expasy.org/protparam/) [58]. The online tool GSDS was used to show the gene structure of *CsbZIP* genes (http://gsds.cbi.pku.edu.cn/). The conserved motif was predicted by the MEME program with a maximum number of motifs 20 (between 10 to 50 optimum width; http://meme-suite.org/) [59]. Then, MEGA 7 software was used to construct the phylogenetic tree of three species by the neighbour-joining method (1000 bootstrap replicates, http://www.megasoftware.net). WoLF PSORT (https://wolfpsort.hgc.jp/) were used to predict the subcellular locations of *C. songorica* bZIP proteins.

### Synteny analysis and chromosome localization

The following three steps were used to identify the paralogous and orthologous gene pairs, 1) OrthoMCL software V5 was used with default settings [60]; 2) BLASTP was used to multiple sequence alignment (E < 1e^− 5^, top 3 matches) within the *C. songorica* genome and between the *C. songorica* and rice genomes; 3) MCL software was used to cluster pairs in to OrthoMCL groups (values for finding clustering of different granularity = 1). The Circos (Circos 0.69) program was used to show the gene pairs [61]. The synonymous (Ks) and non-synonymous (Ka) nucleotide substitutions between paralogous and orthologous gene pairs were calculated based on the comparative synteny map of the *C. songorica* genome and between the rice and *C. songorica* genomes, using Clustal W, PAL2NAL [62] and the yn00 program of the bio-pipeline (https://github.com/tanghaibao/bio-pipeline/tree/master/synteny-pipeline).

### Identification of putative *cis*-elements and LTR retrotransposon in *CsbZIP* genes

The 1500 bp upstream sequences of the *CsbZIP* genes were used to identify the *cis*-elements in the promoter regions with the PlantCARE [63] website (http://bioinformatics.psb.ugent.be/webtools/plantcare/html/).

LTR-FINDER (1.0.5 version) was used to identify the LTR retrotransposon in *CsbZIP* genes (http://tlife.fudan.edu.cn/ltr_finder/) [64].

### Stress treatment and transcriptomic data analysis

Four-week-old seedlings of *C. songorica* were grown in a growth chamber that was maintained at 16 h light 28 °C/8 h dark 24 °C, with an irradiance of 150 μmol quanta m^− 2^ s^− 1^ and 65% relative humidity.

Each pot was filled with a sand/vermiculite (1/1, v/v) mixture, with 0.45 kg per pot. Each plant was irrigated with 100 mL Hoagland nutrient solution every 3 days. The 9-week-old seedlings were treated with 40 °C (heat), 4 °C (cold), 50 mM NaCl (light salt stress), 100 mM NaCl (moderate salt stress), 200 mM NaCl (severe salt stress) and 100 μM ABA (ABA stress). The shoots and roots of each plant were collected 24 h after treatment application in the growth medium, immediately frozen in liquid nitrogen, and stored at − 80 °C. Transcriptome data of *CsbZIP* genes under drought stress were obtained from our previous study [65].

Total RNA of *C. songorica* shoot and root under abiotic stresses were extracted with TRIzol reagent (Invitrogen, USA). The library preparation and deep sequencing were performed by the Novogene Bioinformatics Technology Cooperation (Beijing, China). Sequencing libraries were generated using NEBNext® Ultra™ RNA Library Prep Kit for Illumina® (NEB, USA) according manufacturer’s recommendations. The libraries were then sequenced on a HiSeq2500 with a sequencing read length of 125 bp. Clean reads were obtained by removing reads containing adapters, reads containing poly-N and lower quality reads from the raw reads. The clean reads were mapped to the *C. songorica* genome (data not published) using HISAT2 from the BMK Cloud server (www.biocloud.net). Quantification of gene expression levels were estimated by fragments per kilobase of transcript per million fragments (FPKM) mapped using StringTie (1.3.1) in each sample [66, 67]. DESeq R package (1.10.1) provide statistical routines for determining differential expression in digital gene expression data using a model based on the negative binomial distribution. The resulting FDR (false discovery rate) were adjusted using the PPDE (posterior probability of being DE). Genes with an adjusted FDR < 0.01 and |log2 (foldchange) ≥1 found by DESeq were assigned as differentially expressed.

Finally, a heat map of the *CsbZIP* genes expression profile was shown by the OmicShare tools, a free online platform for data analysis (http://www.omicshare.com/tools). A Venn diagram was generated by the jvenn website (http://jvenn.toulouse.inra.fr/app/example.html).

### Expression profiles of *CsbZIP* genes in different tissues of *C. songorica*

Transcription analysis were performed to identify expression patterns in different tissues, including leaves, roots, seeds, chasmogamous (CH) flowers and cleistogamous (CL) flowers. Roots and leaves samples were obtained from 6-week-old *C. songorica* seedings. Seed samples were mixed with imbibed seeds and germinated seeds. The samples of CH flowers, CL flowers were collected at the green anther stage of *C. songorica*. The methods of RNA-seq and data analysis were the same as described previously.

### Co-expression network construction and enrichment analysis

The co-expression network analysis of *CsbZIP* genes involved in CL flower development and stress response were performed with weighted gene co-expression network (WGCNA; https://horvath.genetics.ucla.edu/html/CoexpressionNetwork/Rpackages/WGCNA/Tutorials/) analysis. For abiotic stress analysis, 59 RNA-seq data were used for WGCNA analysis. For CL flower development, 14 RNA-seq data were used for WGCNA analysis, including CL flowers and CH flowers. The co-expression network was shown by Cytoscape (v 3.5.1; https://cytoscape.org/) software. The co-expression genes were annotated using KOBAS 3.0 (http://kobas.cbi.pku.edu.cn/download.php).

### Quantitative real-time (RT) PCR

Total RNA was isolated from *C. songorica* shoots after stress treatments for qRT-PCR using RNAiso reagent (TaKaRa, Dalian, China). qRT-PCR was performed using SYBR Premix Ex TaqTM (TaKaRa). Approximately 1 μg of RNA was reverse-transcribed into first-stand cDNA with the PrimeScript® RT reagent Kit (TaKaRa), and the product was used as a template for qRT-PCR with specific primers (Additional file 14: Table S10). *CsGAPDH* was used as the reference gene. The relative expression levels were calculated by the comparative CT method. All reactions were performed in triplicate.

## Supplementary information

**Additional file 1: Table S1.** Basic characteristic of *CsbZIP* genes in *C. songorica*.

**Additional file 2: Figure S1.** Gene structure and conserved motif of *CsbZIP* genes based on the evolutionary relationship. a Gene structure analyses were presented with GSDS. The blue boxes indicate upstream / downstream, the orange boxes represent exons, and the black lines indicate introns. b All motifs were identified by MEME database with the complete amino acid sequences of *CsbZIPs*. Each motif was showed by different colored block, with their numbers in the center of the motifs. The number in boxes (1–20) represents motif 1 - motif 20, respectively. The position and length of each colored box represents the actual motif size. The evolutionary tree was carried out with MEGA7.

**Additional file 3: Figure S2.** The major motifs identified by MEME in the putative *CsbZIP* proteins.

**Additional file 4: Figure S3.** Gene location of *bZIP* genes in *C. songorica*. a Statistical analysis of amino acid residues, grand average of hydropathicity, isoelectric points and molecular weight of bZIP genes in *C. songorica*. b The distribution of bZIP genes on *C. songorica* chromosomes, shown as percentages.

**Additional file 5: Table S2.** Synteny blocks of bZIP genes within *C. songorica* genome.

**Additional file 6: Table S3.** Synteny blocks of bZIP genes between *C. songorica* and rice genome.

**Additional file 7: Table S4.** Cis-elememts analysis of *CsbZIP* genes.

**Additional file 8: Table S5.** Expression pattern of CsbZIP genes in different organ.

**Additional file 9: Table S6.** Go enrichment of DEG between CH and CL flowers.

**Additional file 10 Table S7.** Gene list and Go annotation of co-expression genes with selected *CsbZIP* gene involved in CL flower development.

**Additional file 11: Figure S4.** GO enrichment analysis of co-expression genes with *CsbZIP20*, *CsbZIP57*, *CsbZIP59*, *CsbZIP82*.

**Additional file 12: Table S8.** Transcriptome data of *CsbZIP* genes in shoot and root under abiotic stress.

**Additional file 13: Table S9.** Gene list and GO enrichment analysis of co-expression genes with abiotic related genes.

**Additional file 14: Table S10.** Primer list for gene specific primers.

## Data Availability

The datasets generated and analysed during the current study are not publicly available due to the whole genome sequencing work of *C. songorica* has not been published but are available from the corresponding author on reasonable request.

## Abbreviations

bZIP: Basic leucine zipper
CH: Chasmogamous
CL: Cleistogamous
Ka: The rate of nonsynonymous substitutions
Ks: The rate of synonymous substitutions
qRT-PCR: Quantitative real-time reverse transcription PCR
TFs: Transcription factors
WGCNA: Weighted gene co-expression network
