# Supplementary figures and images for: Comprehensive analysis of bZIP transcription factors uncovers their roles during dimorphic floret differentiation and stress response in Cleistogenes songorica
Source: BMC Genomics. 2019 Oct 22;20:760. doi: 10.1186/s12864-019-6092-4 (PMC6805689; doi:10.1186/s12864-019-6092-4)

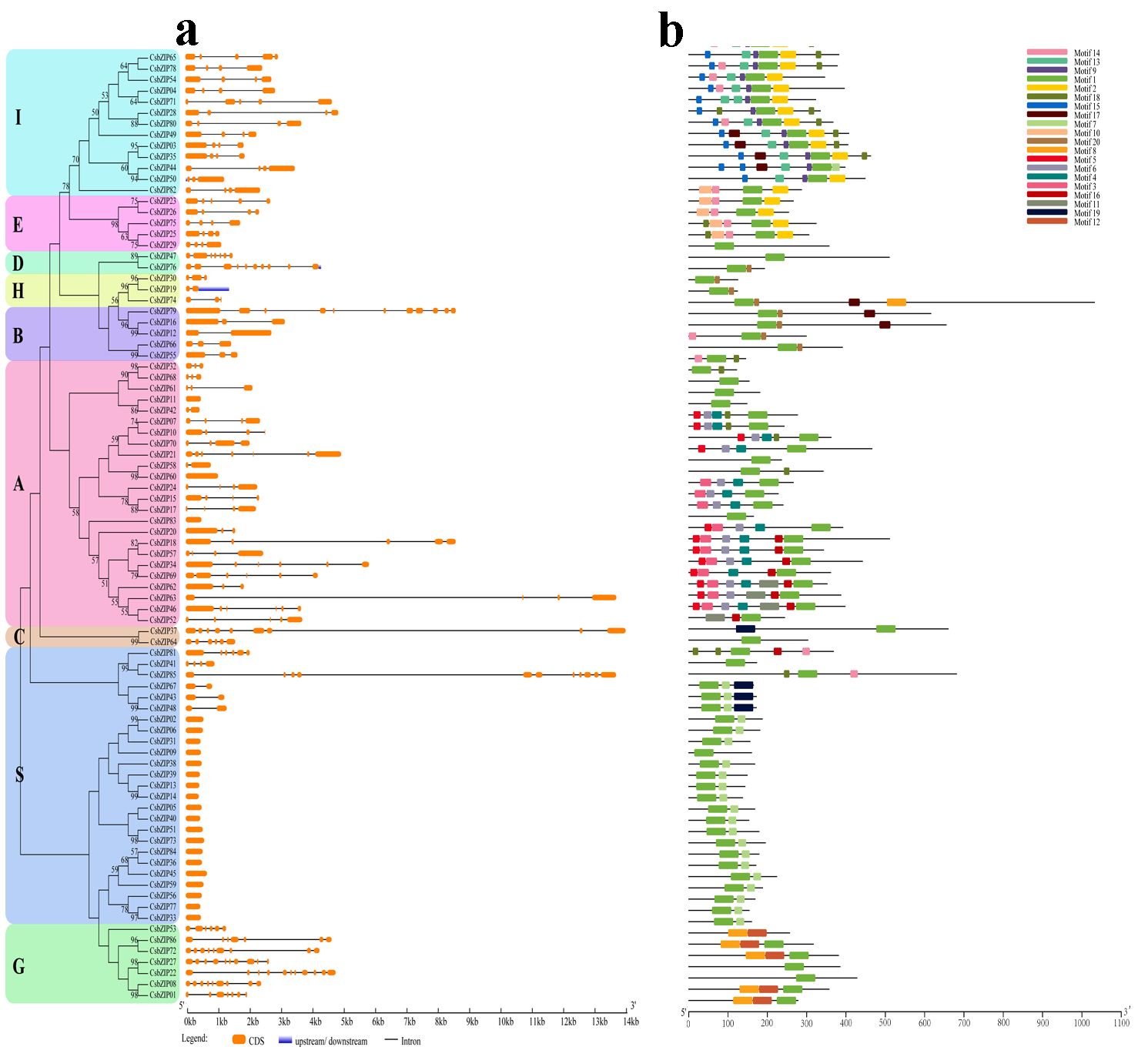

Supplement: Supplementary file 2 — Additional file 2: Figure S1. Gene structure and conserved motif of CsbZIP genes based on the evolutionary relationship. a Gene structure analyses were presented with GSDS. The blue boxes indicate upstream / downstream, the orange boxes represent exons, and the black lines indicate introns. b All motifs were identified by MEME database with the complete amino acid sequences of CsbZIPs. Each motif was showed by different colored block, with their numbers in the center of the motifs. The number in boxes (1–20) represents motif 1 - motif 20, respectively. The position and length of each colored box represents the actual motif size. The evolutionary tree was carried out with MEGA7. [file 12864_2019_6092_MOESM2_ESM.tif]

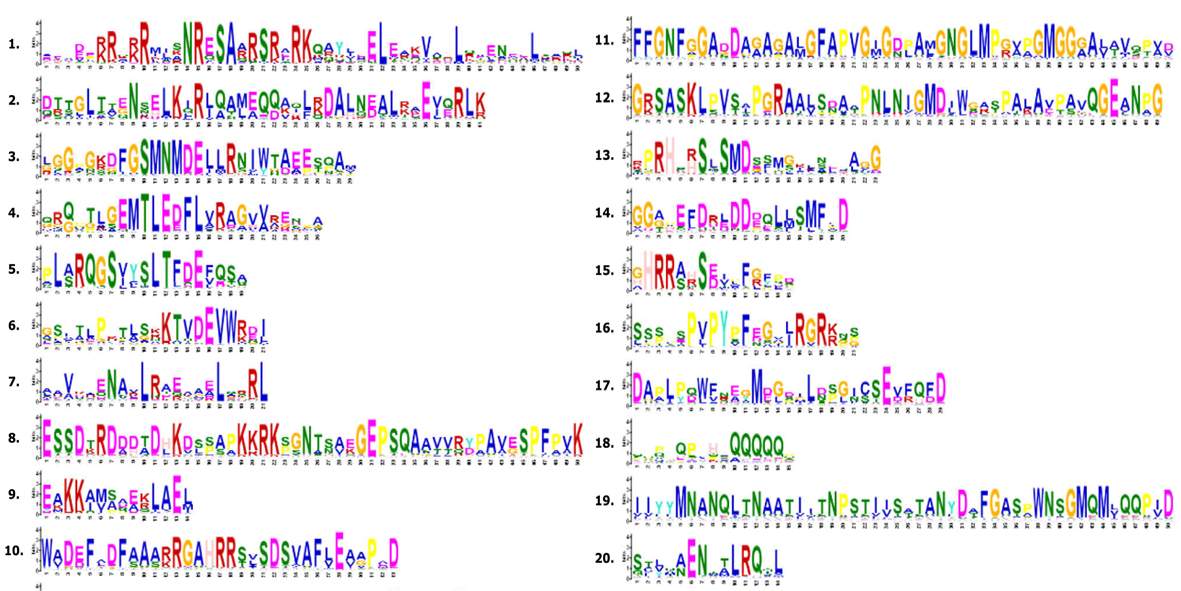

Supplement: Supplementary file 3 — Additional file 3: Figure S2. The major motifs identified by MEME in the putative CsbZIP proteins. [file 12864_2019_6092_MOESM3_ESM.tif]

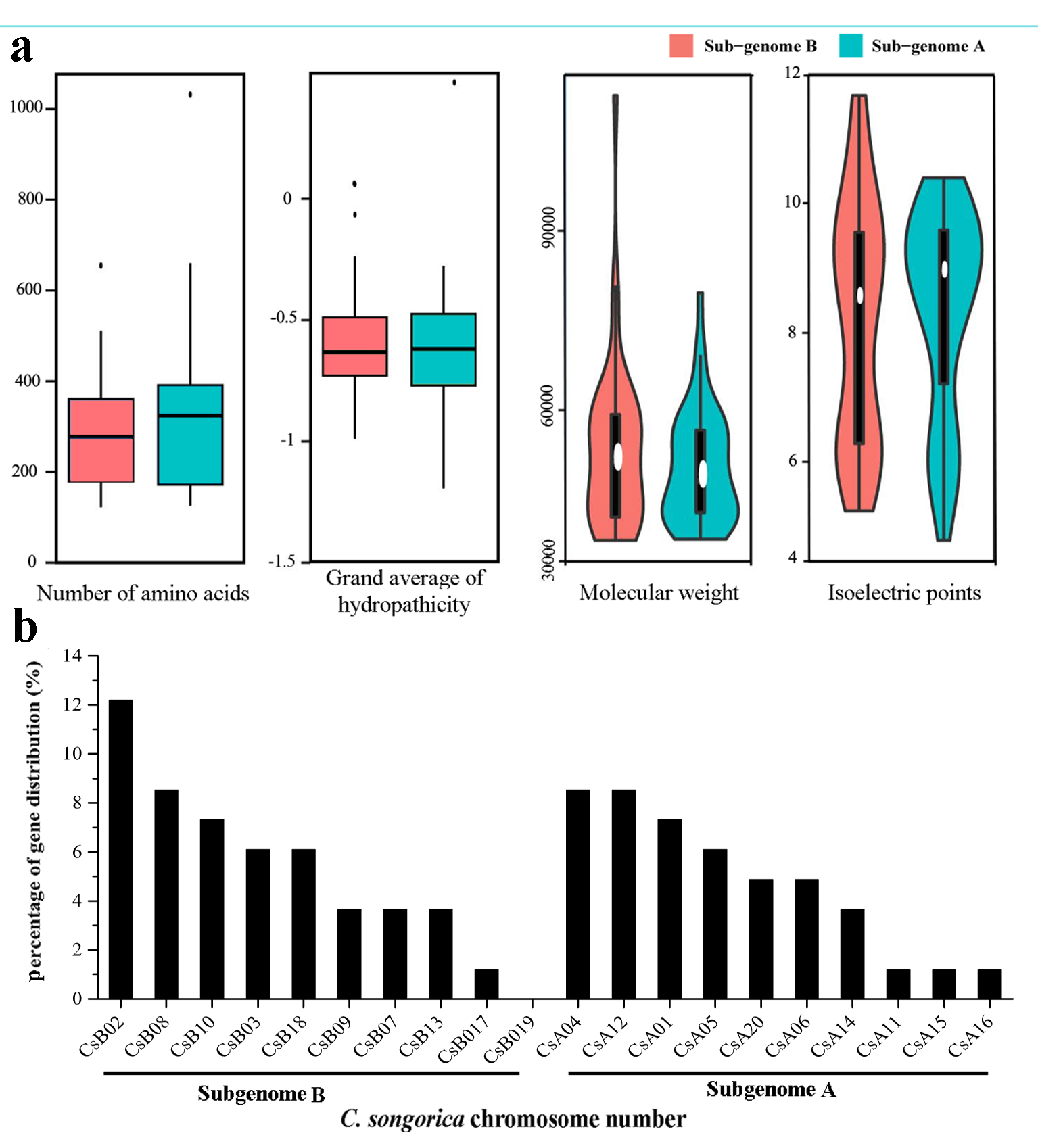

Supplement: Supplementary file 4 — Additional file 4: Figure S3. Gene location of bZIP genes in C. songorica. a Statistical analysis of amino acid residues, grand average of hydropathicity, isoelectric points and molecular weight of bZIP genes in C. songorica. b The distribution of bZIP genes on C. songorica chromosomes, shown as percentages. [file 12864_2019_6092_MOESM4_ESM.tif]

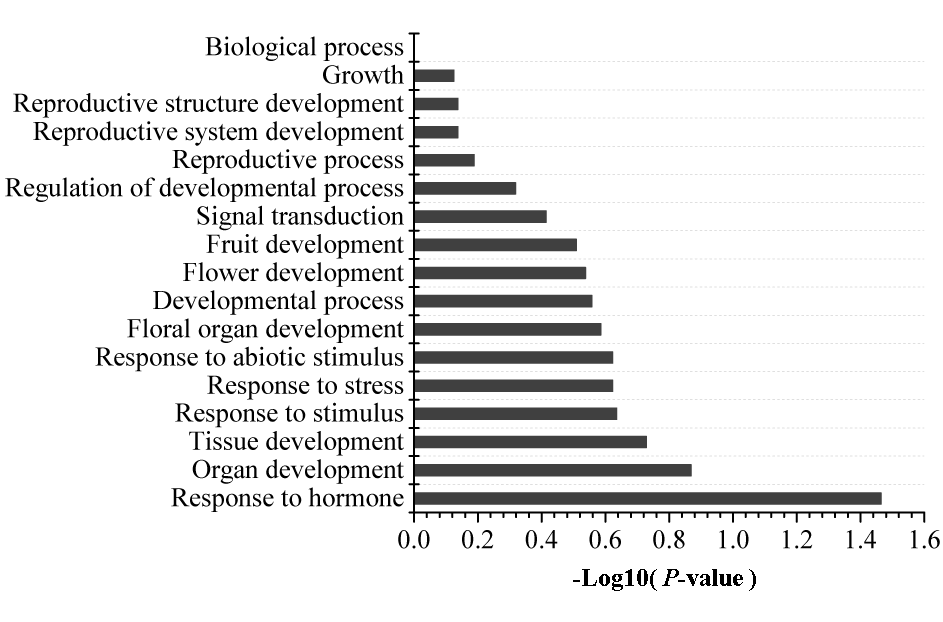

Supplement: Supplementary file 11 — Additional file 11: Figure S4. GO enrichment analysis of co-expression genes with CsbZIP20, CsbZIP57, CsbZIP59, CsbZIP82. [file 12864_2019_6092_MOESM11_ESM.tif]
